# Inverse similarity and reliable negative samples for drug side-effect prediction

**DOI:** 10.1186/s12859-018-2563-x

**Published:** 2019-02-04

**Authors:** Yi Zheng, Hui Peng, Shameek Ghosh, Chaowang Lan, Jinyan Li

**Affiliations:** Advanced Analytics Institute, FEIT, University of Technology Sydney, 15 Broadway, Ultimo, NSW 2007 Australia

**Keywords:** Side-effect prediction, Drug similarity integration, Reliable negative samples, Chemical structure, Target protein

## Abstract

**Background:**

In silico prediction of potential drug side-effects is of crucial importance for drug development, since wet experimental identification of drug side-effects is expensive and time-consuming. Existing computational methods mainly focus on leveraging validated drug side-effect relations for the prediction. The performance is severely impeded by the lack of reliable negative training data. Thus, a method to select reliable negative samples becomes vital in the performance improvement.

**Methods:**

Most of the existing computational prediction methods are essentially based on the assumption that similar drugs are inclined to share the same side-effects, which has given rise to remarkable performance. It is also rational to assume an inverse proposition that dissimilar drugs are less likely to share the same side-effects. Based on this inverse similarity hypothesis, we proposed a novel method to select highly-reliable negative samples for side-effect prediction. The first step of our method is to build a drug similarity integration framework to measure the similarity between drugs from different perspectives. This step integrates drug chemical structures, drug target proteins, drug substituents, and drug therapeutic information as features into a unified framework. Then, a similarity score between each candidate negative drug and validated positive drugs is calculated using the similarity integration framework. Those candidate negative drugs with lower similarity scores are preferentially selected as negative samples. Finally, both the validated positive drugs and the selected highly-reliable negative samples are used for predictions.

**Results:**

The performance of the proposed method was evaluated on simulative side-effect prediction of 917 DrugBank drugs, comparing with four machine-learning algorithms. Extensive experiments show that the drug similarity integration framework has superior capability in capturing drug features, achieving much better performance than those based on a single type of drug property. Besides, the four machine-learning algorithms achieved significant improvement in macro-averaging F1-score (e.g., SVM from 0.655 to 0.898), macro-averaging precision (e.g., RBF from 0.592 to 0.828) and macro-averaging recall (e.g., KNN from 0.651 to 0.772) complimentarily attributed to the highly-reliable negative samples selected by the proposed method.

**Conclusions:**

The results suggest that the inverse similarity hypothesis and the integration of different drug properties are valuable for side-effect prediction. The selection of highly-reliable negative samples can also make significant contributions to the performance improvement.

**Electronic supplementary material:**

The online version of this article (10.1186/s12859-018-2563-x) contains supplementary material, which is available to authorized users.

## Background

Drug side-effects refer to secondary phenotypic responses of the human organisms to drug treatments [1]. They have gained broad public attention because they cause significant fatality and severe morbidity. In America, it is estimated that side-effects are the fourth leading cause of death which should be responsible for 100,000 deaths every year [2]. Moreover, drug side-effects account for about one-third of drug failures during the drug development process [3]. Therefore, it is of critical importance to detect side-effects as early as possible.

Conventional approaches to side-effect prediction during the drug development process are pharmacology assays such as in vivo assays and in vitro assays [4]. However, such experimental predictions are expensive, time-consuming, and tedious. Recently, several computational methods have been proposed to tackle the side-effect prediction problem based on drug profiles [2, 5–15]. These methods can be categorized into target protein-based methods and chemical structure-based methods.

The main idea of target protein-based methods is to relate drug side-effects to drug target proteins directly or indirectly. Previous studies on drug target identification by side-effects have demonstrated the strong associations between drug targets and drug side-effects [16–18]. Yamanishi et al. [5] explored to predict drug side-effects by integrating target proteins and drug structures in a unified framework. Their experiments showed that the prediction accuracy can be improved owing to the integration of target protein information. Researchers developed prediction methods based on pathways which indirectly involve proteins targeted by drugs [7]. Links between pathways and side-effects have been established by analyzing compounds which share the same toxic phenotypes and comparing these pathways with pathways modulated by nontoxic compounds [6]. An efficient algorithm, named CoopeRativE Pathway Enumerator, was proposed to enumerate cooperative pathways which share common active conditions. Then these cooperative pathways were further adopted to predict side-effects and the method achieved satisfactory performance [7].

The principle of chemical structure-based methods is to relate drug side-effects to drug chemical structures. Early in 2007, Bender et al. [8] attempted to predict drug side-effects across hundreds of categories from their chemical structures alone and established correlations between the chemical space and side-effect prediction. Hammann et al. [9] used a decision tree to determine the chemical, physical and structural properties of drugs that predispose them to cause side-effects. Canonical correlation analysis (CCA) was employed for simultaneous prediction of multiple side-effects from chemical structures by Atias [10]. Based on CCA, an improved method called sparse canonical correlation analysis (SCCA) [2] was designed to predict potential drug side-effects from chemical substructures. Related comparison experiments with CCA have demonstrated that SCCA could provide more selective and informative correlation between drug chemical substructures and side-effects without losing performance. Schiber et al. [11] tried a global linkage analysis to map drug chemical features to side-effects on a large scale. However, the authors just aimed to relate chemical structures to side-effects instead of understanding the mechanism of relations. Moreover, they did not provide a framework to predict drug side-effects for drugs.

Usually for the side-effect prediction task, only positive samples which are composed of drugs known to have certain side-effects are validated. There is no definite prior knowledge that a drug is certain not to have a side-effect. Thus there are no validated negative samples in this task. Most existing side-effect prediction methods are based on the closed-world assumption, taking labeled drugs as positive samples and unlabeled drugs as negative samples to perform the prediction directly [2, 5, 8, 15, 19, 20]. Labeled and unlabeled drugs are drugs which are known and not known to have the side-effect respectively according to the prior knowledge. They solely depend on the use of known drug-side-effect relations (i.e., validated positive samples) to make predictions. However, the unlabeled drugs still have considerable probability to have these side-effects. It means that the assumed negative samples may include a considerable number of real positive samples which are yet unknown. As a result, the quality of their negative samples can not be guaranteed, and inaccurate selection of negative samples would largely degrade the prediction performance [21]. To improve the prediction performance, we need good methods to select reliable negative samples for the prediction task.

Existing side-effect prediction methods are essentially based on the assumption that similar drugs are inclined to share the same side-effects [15, 20], which have generated remarkable performance. Thus, it is rational to determine potential negative samples based on the inverse proposition that dissimilar drugs are less likely to share the same side-effects. Based on this hypothesis, we proposed a novel method to select highly-reliable negative samples based on the comprehensive drug similarity from the set of unlabeled drugs. The comprehensive drug similarity measurement integrates the chemical space of drug chemical structures, biological space of drug target proteins and other space of drug substituents, and therapeutic classification into a unified framework to capture drug features from different perspectives. Candidate negative drugs which have lower similarity scores with validated positive drugs were preferentially selected to form the negative drug sample set. We highlight our contributions as follows:

- Development of a drug similarity integration framework which integrates drug chemical structure similarity, drug target protein similarity, drug substituent similarity, and drug therapeutic similarity into a unified comprehensive similarity.
- Selection of reliable negative drug samples for each side-effect from the candidate negative drugs (i.e., unlabeled drugs) based on their comprehensive similarities with validated positive drugs.
- Investigation on the impact of the imbalance between negative samples and positive samples in the training set by comparing against a self-built balanced training set.
- Use of machine learning methods to predict potential drug side-effects based on validated positive drug samples and the selected reliable negative drug samples, and compare the results with the existing predictive methods.
- Extensive comparison experiments demonstrate that side-effect prediction using reliable negative drug samples selected based on the proposed drug similarity integration framework can achieve the best performance.

## Materials

### Drug side-effect profiles

**Fig. 1:**
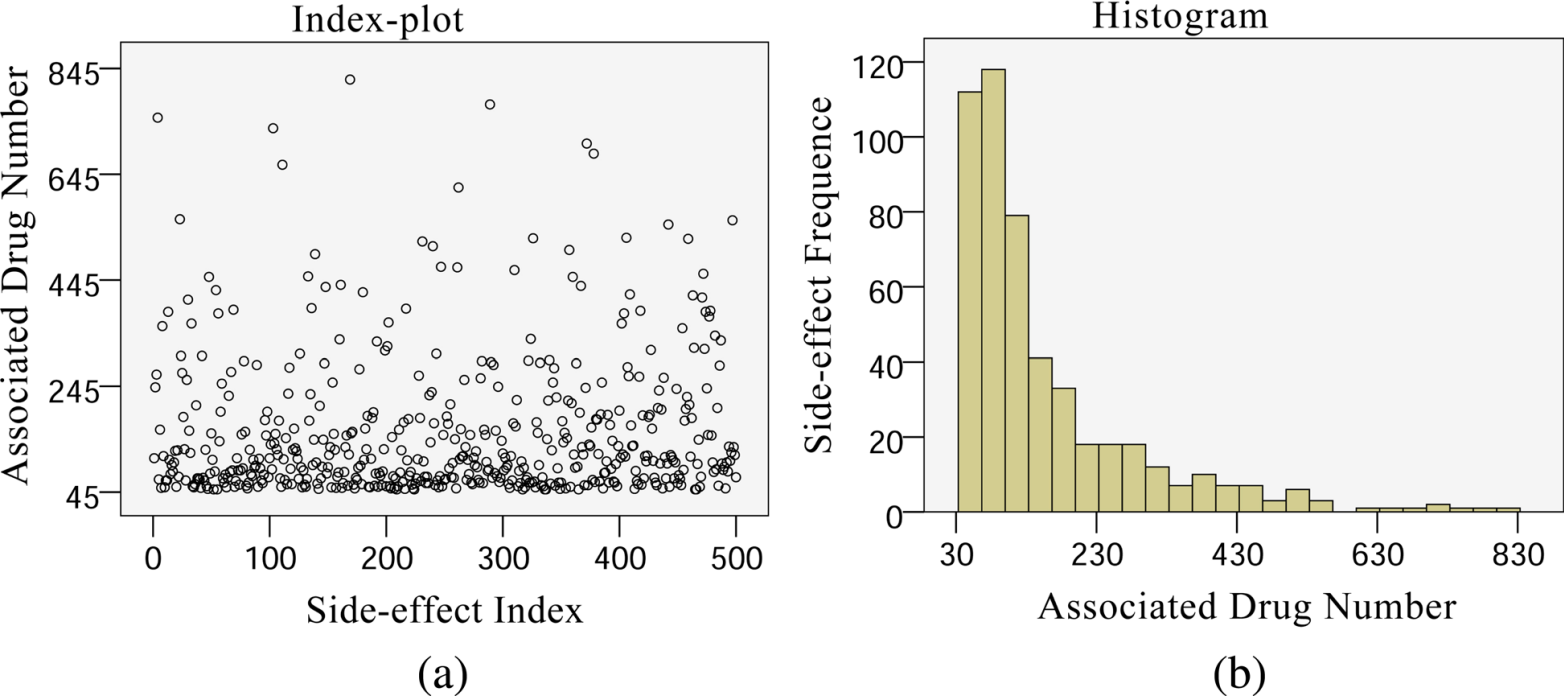
Characteristics of side-effects and their associated drugs. The left panel (**a**) is the index-plot of the number of associated drugs for each side-effect and the right panel (**b**) is the histogram of the associated drug number for the side-effects

The side-effect data set was downloaded from SIDER (version 4.1), a comprehensive drug side-effect database [22]. In this work, we focus on side-effects of drugs which are grouped as “Small Molecules” in the DrugBank database [23, 24]. Our basic idea lies in predicting drug side-effects according to drug similarities. Therefore, those drugs whose similarity information are not available were removed. Correlated with drugs in SIDER, we finally obtained a data set of 917 drugs, 500 side-effect terms, and 78,855 drug side-effect pairs (DSPs). Details of the dataset are described in both Table 1 and Fig. 1. All the above data and the source codes are included in Additional file 1.

**Table 1:** The drug side-effect dataset

Field	Value
Drug group	Small molecules
Number of drugs	917
Number of side-effects	500
Number of DSPs	78,855
Average side-effects per drug	86.0
Average drugs per side-effect	157.7

### Drug similarities

#### Chemical structure similarity

Chemical structures of drug molecules were downloaded from DrugBank (stored in SMILES files) [23, 24]. The molecular fingerprints were retrieved from these SMILES files using an open source tool called Chemical Development Kit (CDK) [25]. The chemical structure similarity score between two drugs is calculated according to the Tanimoto 2D score between their molecular fingerprints. For a drug *d*, it can be represented by its hybridization fingerprint $f^{d} \left (f_{i}^{d} \in \{0,1\}, i\in \{1...1024\}\right)$. Then the chemical similarity score between drug *d*_*j*_ and drug *d*_*k*_ is given by:

1 $$ S_{chem}\left(d_{j},d_{k}\right)=Tanimoto\left(f^{j},f^{k}\right)=\frac{{\sum}_{l=1}^{1024}\left(f_{l}^{j}\land f_{l}^{k}\right)}{{\sum}_{l=1}^{1024}\left(f_{l}^{j}\lor f_{l}^{k}\right)},  $$

where ∧ and ∨ are bit-wise “and” and “or” operators respectively; $f_{l}^{j}$ and $f_{l}^{k}$ are the *l*^*th*^ bit of fingerprints of drug *d*_*j*_ and drug *d*_*k*_ respectively.

#### Drug target protein similarity

The similarity of two proteins is calculated based on the overlapping rate of their associated Gene Ontology (GO) terms. Suppose *GO*^*m*^ and *GO*^*n*^ are the GO term sets for protein *p*_*m*_ and protein *p*_*n*_ respectively, the similarity score between *p*_*m*_ and *p*_*n*_ is defined as

2 $$ S_{go}\left(p_{m},p_{n}\right)=\frac{GO^{m} \cap GO^{n}}{GO^{m} \cup GO^{n}},  $$

where ∩ and ∪ are “intersection” and “union” operators respectively. The GO terms of target proteins were downloaded from the EMBL-EBI website [26, 27]. Drugs are expected to interact with proteins which have similar cellular components, or share similar molecular functions, or go through similar biological processes. Therefore, all the three types of ontologies were utilized in the similarity definition. Then the drug target protein similarity between each pair of drugs was calculated by integrating protein similarities of their target proteins. The target protein similarity score between drug *d*_*j*_ and drug *d*_*k*_ is calculated as follows:

3 $$ S_{tar}\left(d_{j},d_{k}\right)=\frac{{\sum}_{m=1}^{N_{j}}{\sum}_{n=1}^{N_{k}}S_{go}\left(p_{m},p_{n}\right)}{N_{j}*N_{k}},  $$

where *N*_*j*_ and *N*_*k*_ are the total number of proteins in the interacted protein sets of drug *d*_*j*_ and drug *d*_*k*_ respectively.

#### Drug substituent similarity

Substituents are atoms which replace hydrogen atoms on the parent chain of hydrocarbon. Its subsets, functional groups, are responsible for the characteristic chemical reactions of molecules. Therefore, it’s reasonable to measure similarity between drugs via their substituents. The drug substituent similarity between drug *d*_*j*_ and drug *d*_*k*_ is calculated via Jaccard score which is given by:

4 $$ S_{sub}\left(d_{j},d_{k}\right)=\frac{{SUB}_{j} \cap {SUB}_{k}}{{SUB}_{j} \cup {SUB}_{k}},  $$

where *SUB*_*j*_ and *SUB*_*k*_ are the substituent sets of drug *d*_*j*_ and *d*_*k*_ respectively; ∩ and ∪ are intersection and union operators respectively.

#### Drug therapeutic similarity

The Anatomical Therapeutic Chemical (ATC) codes of drugs are assigned according to their therapeutic, pharmacological and chemical properties [28]. The ATC codes have been demonstrated to be useful in predicting the drug poly-pharmacological profiles [29]. Hence, we take the ATC codes as one part of the drug similarity measurement. The ATC codes used in this work were extracted from the DrugBank database. There are 5 levels in the ATC code. First, we calculate the drug therapeutic similarity at each level separately. The *l*^*th*^ level drug therapeutic similarity *S*_*l*_ between the drug *d*_*j*_ and *d*_*k*_ is defined as:

5 $$ S_{l}\left(d_{j},d_{k}\right)=\frac{{ATC}_{l}\left(d_{j}\right) \cap {ATC}_{l}\left(d_{k}\right)}{{ATC}_{l}\left(d_{j}\right) \cup {ATC}_{l}\left(d_{k}\right)},  $$

where *ATC*_*l*_(*d*_*j*_) denotes the *l*^*th*^ level ATC code for drug *d*_*j*_; ∩ and ∪ are intersection and union operator respectively. The average value of the five-level similarity scores is used as the therapeutic similarity of a drug pair:

6 $$ S_{thera}\left(d_{j},d_{k}\right)=\frac{{\sum}_{l=1}^{n}S_{l}\left(d_{j},d_{k}\right)}{n},  $$

where *n*=5, is the total number of ATC code levels.

## Methods

The outputs of side-effect prediction are discrete side-effect labels, thus we modelled the side-effect prediction as a classification problem instead of a regression problem. Specifically, we transformed the side-effect-prediction problem into a set of independent binary classification problems, where each drug causes or does not cause a given side-effect. For each side-effect, we built a classifier using its validated positive drugs and selected reliable negative drugs. In this section, the framework to integrate drug similarity is introduced first. Then, processes to select reliable negative samples based on integrated drug similarities are detailed. And finally, we present the way to build classifiers for side-effects.

### Drug similarity integration framework

We integrate the above four measurements of drug similarities into a single comprehensive similarity using three consensus similarity inference methods: maximum, mean and geometric mean.(1) Maximum

7 $$ \begin{aligned} S_{max}\left(d_{j},d_{k}\right)&=max\left\{S_{chem}\left(d_{j},d_{k}\right),S_{tar}\left(d_{j},d_{k}\right),\right.\\ & \quad\qquad\left.S_{sub}\left(d_{j},d_{k}\right),S_{thera}\left(d_{j},d_{k}\right)\right\}, \end{aligned}  $$

(2) Mean

8 $$ \begin{aligned} S_{mean}\left(d_{j},d_{k}\right)&=\left[S_{chem}\left(d_{j},d_{k}\right)+S_{tar}\left(d_{j},d_{k}\right)\right.\\ &\quad+\left.S_{sub}\left(d_{j},d_{k}\right)+S_{thera}\left(d_{j},d_{k}\right)\right]/4, \end{aligned}  $$

(3) Geometric Mean

9 $$ {{} \begin{aligned} S_{GM}\left(d_{j},d_{k}\right)=\\ \sqrt[4]{S_{chem}\left(d_{j},d_{k}\right)\!*\!S_{tar}\left(d_{j},d_{k}\right)*S_{sub}\left(d_{j},d_{k}\right)*S_{thera}\left(d_{j},d_{k}\right)}, \end{aligned}}  $$

where *S*_*chem*_(*d*_*j*_,*d*_*k*_), *S*_*tar*_(*d*_*j*_,*d*_*k*_), *S*_*sub*_(*d*_*j*_,*d*_*k*_) and *S*_*thera*_(*d*_*j*_,*d*_*k*_) are chemical similarity, target protein similarity, drug substituent similarity and drug therapeutic similarity between drug *d*_*j*_ and *d*_*k*_ respectively; *S*_*max*_(*d*_*j*_,*d*_*k*_), *S*_*mean*_(*d*_*j*_,*d*_*k*_) and *S*_*GM*_(*d*_*j*_,*d*_*k*_) are the three combined comprehensive similarities for the drug pair drug *d*_*j*_ and *d*_*k*_.

### Negative sample selection based on comprehensive drug similarities for side-effect predictions

**Fig. 2:**
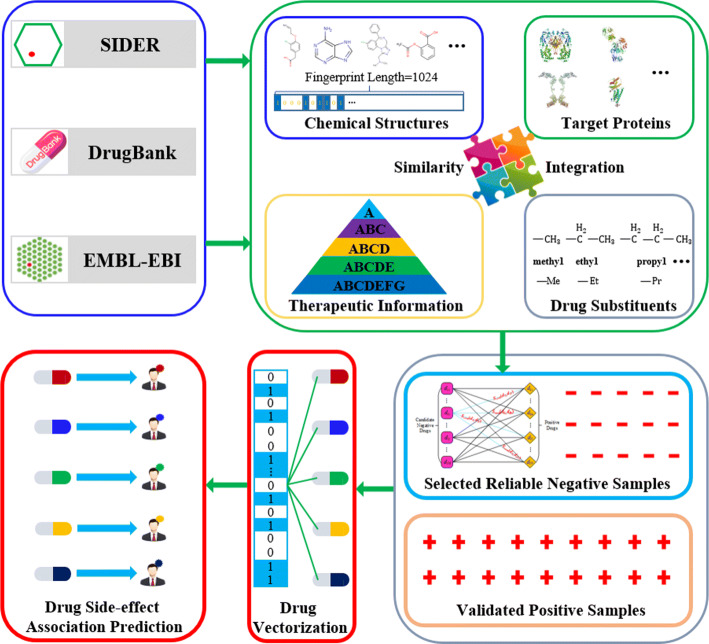
Flow diagram of drug side-effect prediction with the proposed negative sample selection method using the comprehensive drug similarity

Most existing prediction methods assume that similar drugs tend to share the same side-effects. We adopt not only this assumption, but also its inverse proposition to make predictions. Particularly, we adopt its inverse proposition, i.e., dissimilar drugs are less likely to share the same side-effects, to select highly-reliable negative samples. Figure 2 illustrates the flow diagram of the proposed method to select negative samples for predictions.

Starting with the preprocessing procedure, side-effects and drugs which do not meet the requirements are removed (see “Materials” section). Then we compute the comprehensive similarity between every two drugs from the 917 drugs. Following that, we build the positive drug set and the negative drug set for each side-effect. Here we take the side-effect *se* as an example to describe the process.

- Build the positive drug set and the candidate negative drug set for *se*. Specifically, the positive drug set and the candidate negative drug set are formed by drugs which are known and unknown to cause *se* in the prior knowledge respectively.
- Calculate the accumulative similarity score for each drug in the candidate negative drug set using the comprehensive drug similarity.
  **Example.** The accumulative similarity score of a candidate negative drug *d*_*c*,*i*_ equals the sum of similarities between *d*_*c*,*i*_ and each drug in the positive drug set.
  10 $$ \begin{aligned} {Score}_{d_{c,i}} = \sum\limits_{j=1}^{N} S_{com}\left(d_{c,i}, d_{p,j}\right), \end{aligned}  $$
  where *S*_*com*_∈{*S*_*max*_,*S*_*mean*_,*S*_*GM*_}, is the comprehensive similarity; *d*_*p*,*j*_ is the *jth* drug in the positive drug set (1≤*j*≤*N*); N is the total drug number in the positive drug set.
- Rank all drugs in the candidate negative drug set in an ascending order of their accumulative similarity scores, and those with lower scores are preferably selected to form the negative drug set. The threshold score depends on the number of negative drugs should be selected.

After we get the validated positive drug set and selected negative drug set for all side-effects, we vectorize each drug as a 917-dimensional feature vector using comprehensive drug similarities. For example, drug *d*_*i*_ is represented as *d*_*i*_={*S*_*com*_(*d*_*i*_,*d*_1_),...,*S*_*com*_(*d*_*i*_,*d*_*j*_),...,*S*_*com*_(*d*_*i*_,*d*_917_)}^*T*^, where each element encodes the comprehensive drug similarity between *d*_*i*_ and each drug from the whole drug set. Finally, we construct one classifier for each side-effect, optimize related parameters via cross-validations, and finally use the optimized classifier to predict potential drug-side-effect associations. The inputs are vectors of the validated positive drug samples and the selected negative drug samples.

## Results and discussions

The prediction results under different similarity measurements and different similarity integration methods are presented. The prediction performances on balanced and imbalanced training sets in terms of positive and negative samples are also compared through a series of experiments. Finally, we demonstrate the excellent prediction performances of approaches achieved by using the highly-reliable negative samples selected based on the inverse similarity hypothesis and the similarity integration framework.

### Performance evaluation metrics

To evaluate the performance of side-effect prediction, 5-fold cross-validation was performed in the following way: (1) positive and negative samples (drugs) are combined to form a gold standard set; (2) drugs in the gold standard set are split into five roughly equal-sized subsets; (3) each subset is used as the test set, and the remaining four subsets are taken as the training set in turn to test and train the predictive models; (4) the final performance is evaluated on all results over 5-folds. Precision, recall, *F*1-score and their macro values are used as performance indicators:

11 $$ Precision=\frac{TP}{TP+FP},  $$

12 $$ Recall=\frac{TP}{TP+FN},  $$

13 $$ F1\text{-}score=\frac{2*Precision*Recall}{Precision+Recall},  $$

14 $$  Macro\_X=\frac{{\sum}_{i=1}^{n}{X_{i}}}{n},  $$

where *TP* and *FP* are the correctly and falsely predicted positive drug number, *FN* is the falsely predicted negative drug number and *X*∈{Precision, Recall, *F*1-score }.

### Evaluation on drug similarity integration framework

**Fig. 3:**
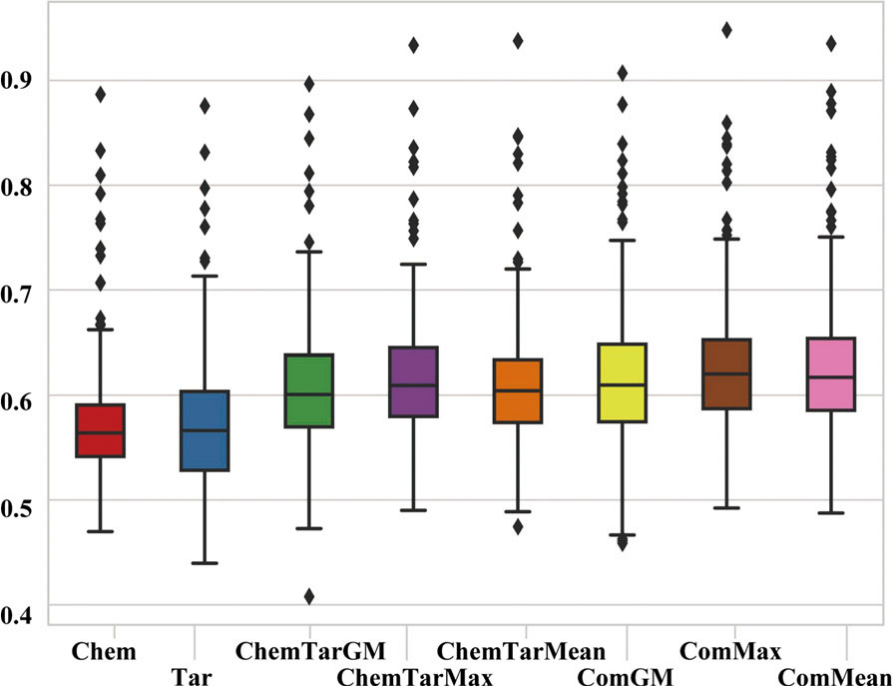
Boxplots of the *F*1-scores for different similarity measurements and different similarity integration methods using the KNN classifier

To demonstrate advantages of the proposed drug similarity integration framework, we report prediction performances under different similarity measurements and different similarity integration methods. We tested eight situations derived from the four similarity measurements and three similarity integration methods: (1) Chem, (2) Tar, (3) ChemTarMax, (4) ChemTarMean, (5) ChemTarGM, (6) ComMax, (7) ComMean and (8) ComGM, where Chem, Tar, ChemTar and Com denote the four similarity measurements, namely the chemical, target and chemical-target and comprehensive similarity respectively; Max, Mean and GM denote the three similarity integration approaches, namely maximum, mean and geometric mean respectively. A typical classifier, K-Nearest Neighbors (KNN), was employed to perform these tasks. The *k* parameter of KNN was set as 50 based on 5-fold cross validation optimization. In addition, all drugs known and unknown to cause the side-effect were directly used as positive and negative samples for prediction. Detailed results of the eight situations are illustrated in Fig. 3.

**Fig. 4:**
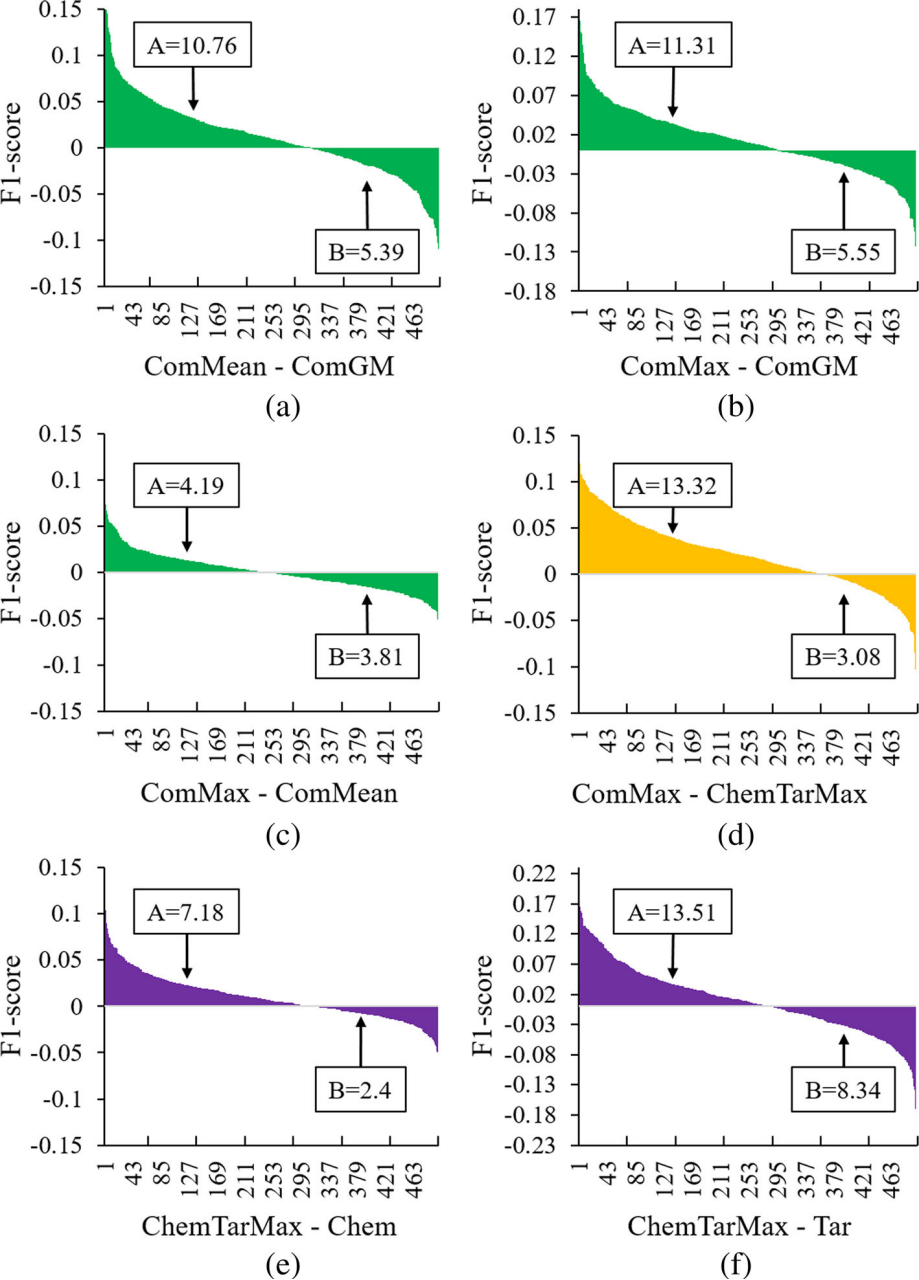
Differences among similarity measurements and similarity integration methods. In each panel, the x-axis denotes the index of each side-effect and the y-axis denotes the *F*1-score difference between two methods. For instance, Fig. 4a describes the differences between “ComMean” and “ComGM” using the *F*1-score of each side-effect from ComMean minus that from ComGM (i.e. difference= *F*1-score(ComMean) −*F*1-score(ComGM)). Thus we can identify which method performs better by comparing the area under the curve above zero (i.e., area A) with the area above the curve under zero (i.e., area B). Panel (**a**) shows the F1-score difference between “ComMean” and “ComGM”; Panel (**b**), (**c**) and (**d**) illustrate the F1-score difference between “ComMax” and “ComGM/ComMean/ChemTarMax” respectively; Panel (**e**) and (**f**) shows the F1-score difference between “ChemTarMax” and “Chem/Tar” respectively

It can be seen that for situations based on chemical-target similarity as well as the comprehensive similarity, the integration method “Mean” and “Maximum” perform much better than “Geometric Mean”, and “Maximum” outperform “Mean” a bit (detailed in Fig. 4a, b and c. Note that Fig. 4 shows differences of F1-scores, not F1-scores as defined). Therefore, we leveraged “Maximum” as the integration method in the subsequent experiments. As for performances of different similarity measurements, chemical-target based situations achieved better performance than chemical-structure based situations and target-protein based situations (see Fig. 4e and f). Situations based on the proposed comprehensive similarity outperformed the chemical-target based situation (see Fig. 4d), producing the best results. Examples of side-effects with low *F*1-scores are “unspecified visual loss (C3665346)”, “hyperphosphataemia (C0085681)”, “corneal opacity (C0010038)”, “red blood cell sedimentation rate increased (C0151632)”, and “exacerbation of asthma (C0349790)”. Examples of high *F*1-score side-effects are “pseudomembranous colitis (C1257843)”, “nausea (C0027497)”, “febrile neutropenia (C0746883)”, “headache (C0018681)”, and “vomiting (C0042963)”. Please refer to the specific *F*1-scores of each situation in Additional file 2: Table S3.

### Evaluation on balanced and imbalanced training set

It can be seen from Fig. 1 that the number of drugs with which a side-effect is associated varies a lot. It means the labeled drugs and unlabeled drugs for most side-effects are imbalanced. In fact, 479 (95.8%) side-effects have more unlabeled drugs than labeled drugs. If all labeled and unlabeled drugs were directly taken as the positive and negative samples respectively to perform the side-effect prediction, the imbalance between them would become a problem which is challenged in the field of machine learning [30]. We investigated the impact of imbalance between positive samples and negative samples on side-effect prediction performance, before reporting results of the proposed negative sample selection method.

To simply compare with the original imbalanced training set, we developed a balanced training set in the following way: (1) the smaller number *n*_*s*_, between the labeled drug number and the unlabeled drug number was obtained; (2) *n*_*s*_ labeled drugs were selected to form the positive drug sample set; (3) *n*_*s*_ unlabeled drugs were selected to form the negative drug sample set. More efficient classifiers, including Extreme Learning Machine (ELM), Support Vector Machine (SVM) and Radial Basis Function (RBF) networks were employed in this task. Their key parameters are optimized as follows: KNN (*k*=50), ELM (active function =sigmoid, hidden neuron number =150), SVM (kernel type =radial basis function, gamma =0.07), RBF (spread =500) (same settings hereinafter). We tested eight situations: (1) KNNComBal, (2) KNNComUnbal, (3) ELMComBal, (4) ELMComUnbal, (5) SVMComBal, (6) SVMComUnbal, (7) RBFComBal and (8) RBFComUnbal. Since the best prediction performance obtained among the three drug similarity integration methods was via “Maximum”, we used it again as the integration method for the eight situations. These situations are named according to the classifier involved, similarity measurements, and balance or not. Situations ending with “Bal” are based on the self-built balanced training set and those ending with “Unbal” are based on the original imbalanced training set. For example, SVMComBal refers to the situation which is performed by SVM based on the self-built balanced training dataset using the comprehensive similarity.

**Fig. 5:**
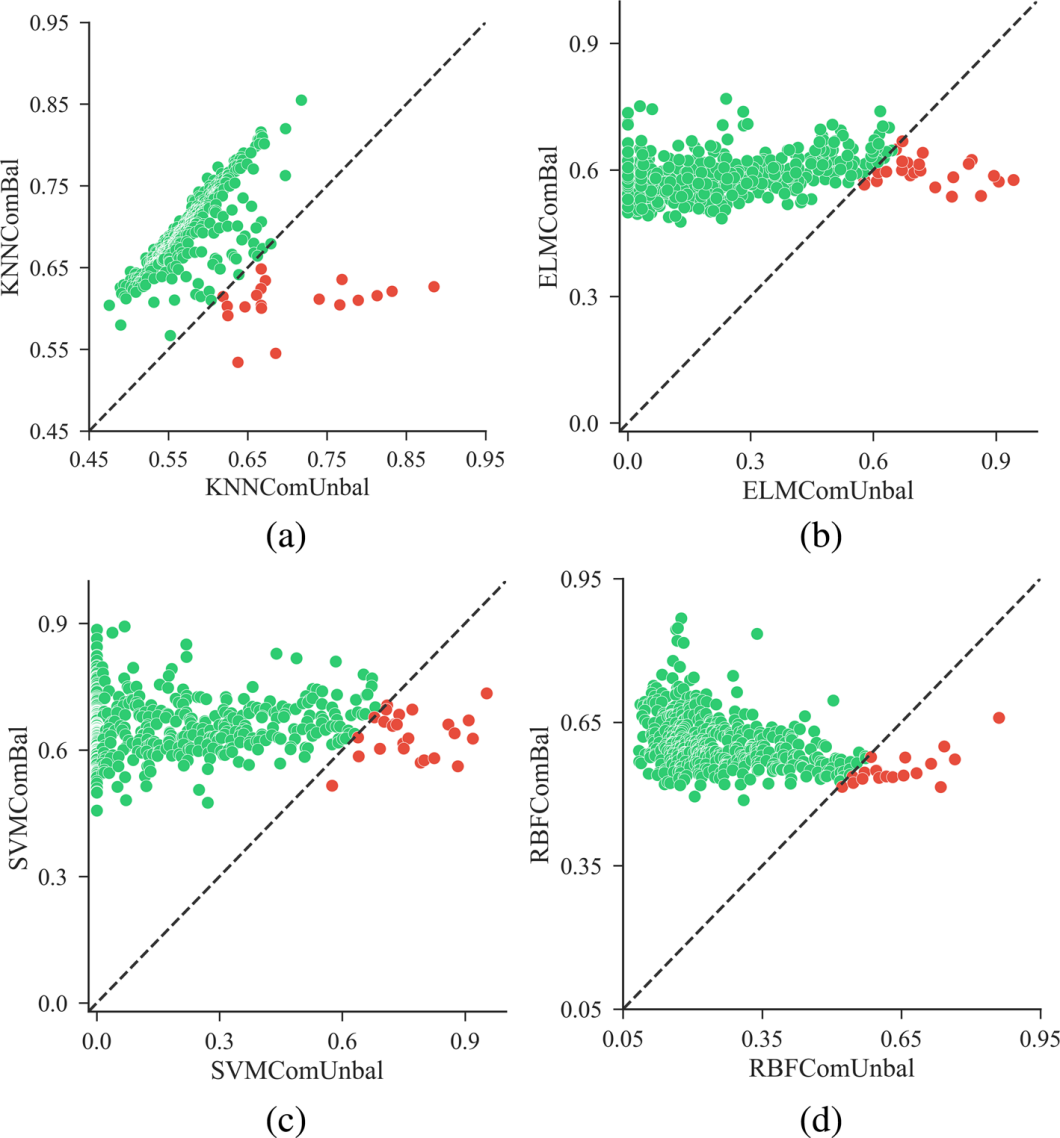
Scatter plots of *F*1-scores for different classifiers using the comprehensive similarity on balanced and imbalanced training sets. The x-axis denotes *F*1-scores of results based on imbalanced training sets, and the y-axis for balanced training sets. The line “ *y*=*x*” on which *F*1-scores are equal, is the reference line to better visualize the results. Panel (**a**), (**b**), (**c**) and (**d**) show the scatter plots of F1-scores on balanced and imbalanced training sets using KNN, ELM, SVM and RBF respectively

To avoid bias, situations based on the self-built balanced training set were repeated 5 times. Each time, the *n*_*s*_ positive drugs and the *n*_*s*_ negative drugs were selected randomly. Average values of the performance evaluation metrics were used for comparison. The scatter plots of prediction results are shown in Fig. 5. From Fig. 5, we can see that most dots are located at the upper side of the reference line for all the classifiers. It reveals that situations based on the balanced training set outperform those on the imbalanced training set for most side-effects.

In Fig. 5b and c, a few dots (i.e., side-effects) are concentrated in the left side of the chart and are close to the *y*-axis, where *F*1-scores of the imbalanced situations are close to 0. It is caused by the large degree of imbalance between the positive and negative samples in the training set. Moreover, ELM and SVM are sensitive to the imbalanced training set. We examined the ratios of positive and negative samples in the imbalanced training sets of these side-effects. For most low *F*1-score side-effects, the number of negative samples in the training set is several times more than positive samples. For example, in terms of side-effects with *F*1-score ≤0.1 using ELMComUnBal, the negative samples are 10.75 times that of positive samples on average. A large degree of imbalance led to the bias of the classification decision boundary against the positive samples, therefore very few samples were predicted as positive drugs. It means the true positive rate is low, resulting in a low *F*1-score.

In Fig. 5b, c and d, a majority of side-effects for which the imbalanced situations outperformed the balanced ones, have relatively high *F*1-scores. Analogously, we investigated the numbers of positive and negative samples for these side-effects. It was found that most of these side-effects have more positive samples in the training set. Taking results from SVMComUnBal as an example, 21 out of 23 side-effects which own higher *F*1-scores than SVMComBal, have more positive samples in the training set.

With the above analyses, it is suggestive that the ratios of positive and negative samples in the training set can influence the prediction performance a lot. Using imbalanced training sets, classifiers are easy to fall into bias. Therefore, situations with balanced training sets can perform better than those situations with imbalanced training sets on most side-effects. This observation can also be confirmed by prediction results using the similarity ChemTar (Additional file 3: Figure S1).

### Performance improvement brought by the selection of highly-reliable negative samples

**Fig. 6:**
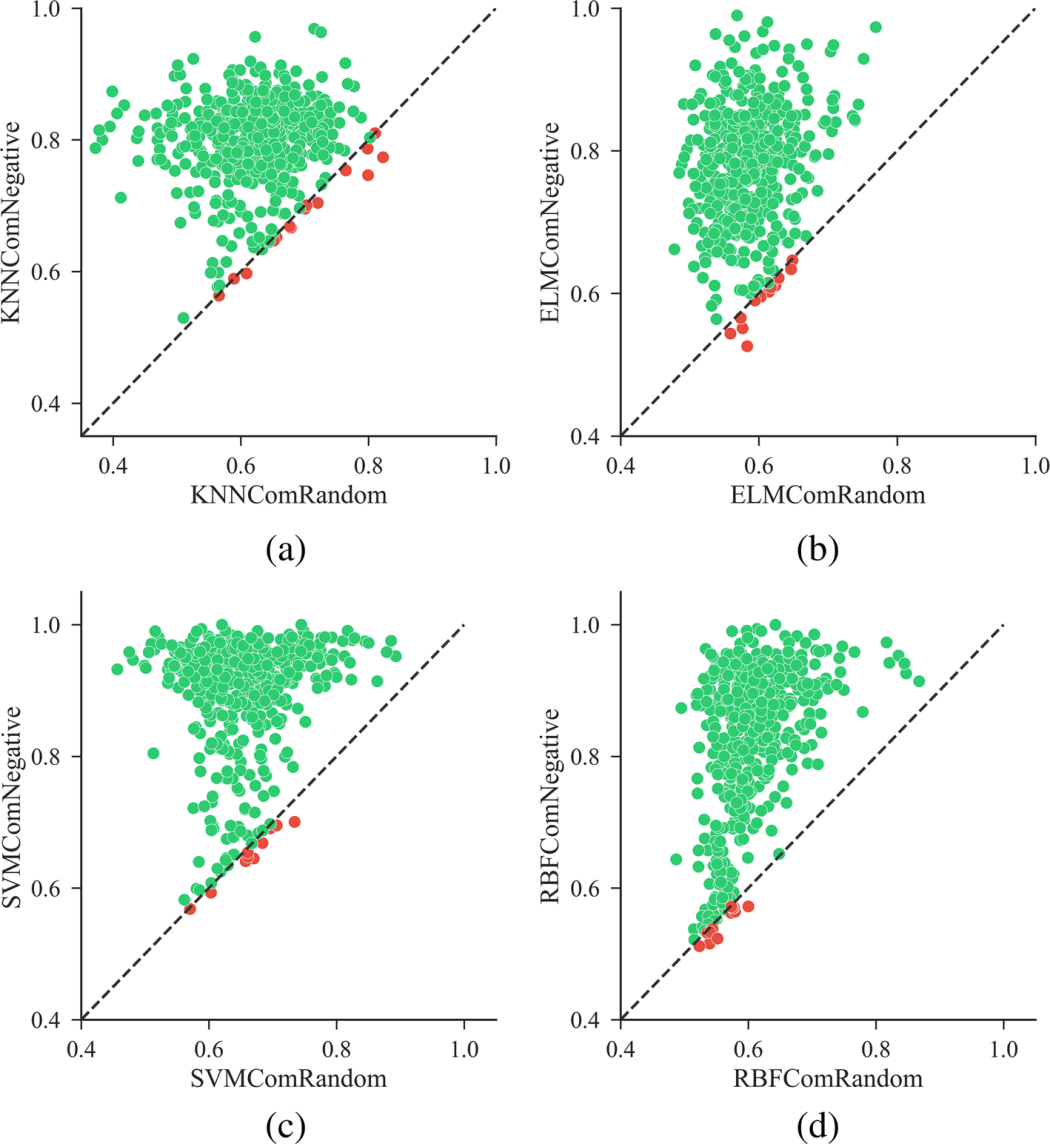
Comparison results using the proposed negative sample selection method and random sample selection method. The x-axis denotes *F*1-scores of results based on negative samples selected randomly, and the y-axis denotes *F*1-scores of results based on negative samples selected by the proposed method. The line “ *y*=*x*” on which *F*1-scores are equal, is used as the reference line. Panel (**a**), (**b**), (**c**) and (**d**) show the scatter plots of F1-scores using the proposed negative sample selection method and random sample selection method achieved by KNN, ELM, SVM and RBF respectively

From analyses presented in the previous sections, it has been understood that different similarity measurements, different similarity integration methods, and the balance ratios between positive and negative samples have heavy influence on the prediction performance. It is confirmed that approaches based on self-built balanced training sets achieved better performance than those based on original imbalanced training sets. With the best options of similarity measurements, integration methods and balance strategy, we evaluated the performance of side-effect prediction when negative samples are selected by the proposed method. As a comparison, we randomly selected negative samples for each side-effect from its unlabeled drugs. We treat this method as the “Baseline”. We tested the following situations: (1) KNNComNegative, (2) KNNComRandom, (3) ELMComNegative, (4) ELMComRandom, (5) SVMComNegative, (6) SVMComRandom, (7) RBFComNegative and (8) RBFComRandom, where situations ending with “Negative” stand for the negative samples selected by the proposed negative sample selection method, while those ended with “Random” denote the negative samples selected randomly. To avoid bias, situations based on randomly selected negative samples were repeated 5 times. Note that all the above situations are based on the comprehensive similarity, the similarity integration method “Maximum”, and the proposed strategy to build balanced training sets (see Evaluation on balanced and imbalanced training set). Related results are illustrated in Fig. 6. As shown in all sub-graphs of Fig. 6, a majority of dots are located at the upper side of each reference line. This suggests that performances of all classifiers were significantly improved owing to the proposed negative sample selection method.

We further investigated the improvement on macro-averaging values of the three performance measurement indices (See Table 2). They were calculated as the average values of all side-effects using equation (14). The highest performance of each classifier was highlighted via bold numbers. Situations using the proposed negative sample selection method based on the comprehensive similarity (ComNegative) achieved significantly higher performance than those using randomly selected negative samples based on comprehensive similarity (ComRandom) or chemical-target similarity (ChemTarRandom, see Additional file 3: Figure S2). For example, for KNN, ELM, SVM, and RBF, the macro-averaging *F*1-score improvement over “ComRandom” is 18.1%, 18.8%, 24.3%, 21.6% respectively, over “ChemTarRandom” is 21.9%, 20.2%, 29.2%, 25.1% respectively.

**Table 2:** Macro-averaging *F*1-score/precision/recall of four typical classifiers based on negative samples selected by the proposed negative sample selection method and randomly selected negative samples

Method	Classifier	Macro_ *F*1	Macro_P	Macro_R
ComNegative	KNN	**0.800**	**0.728**	**0.772**
ComRandom	KNN	0.619	0.598	0.651
ChemTarRandom	KNN	0.581	0.553	0.626
ComNegative	ELM	**0.774**	**0.761**	**0.604**
ComRandom	ELM	0.586	0.572	0.601
ChemTarRandom	ELM	0.572	0.561	0.585
ComNegative	SVM	**0.898**	**0.938**	**0.861**
ComRandom	SVM	0.655	0.670	0.642
ChemTarRandom	SVM	0.606	0.622	0.598
ComNegative	RBF	**0.822**	**0.828**	**0.818**
ComRandom	RBF	0.606	0.592	0.622
ChemTarRandom	RBF	0.571	0.561	0.583

The best performance for each classifier is showed in boldface

Both the results from Fig. 6 and Table 2 confirm the performance improvement brought by the selected highly-reliable negative samples. The proposed method is based on the assumption that drugs dissimilar to any drugs known to cause a given side-effect are less likely to cause the side-effect. Drugs that locate far from all positive samples in the chemobiological space are used as negative samples, which really contributed to improve the prediction performance for different classifiers. The selected high-reliable samples help to learn an optimal classification decision boundary, better differentiating positive samples and negative samples.

### Comparison with other methods

To demonstrate the superior performance of the proposed method, we compared it with several state-of-the-art methods on a widely-used bench-marking dataset (i.e., Liu’s dataset) [15, 20, 31, 32]. There are 832 drugs and 1385 side-effects in this dataset. Since the ATC codes and substituents of some drugs are not available, we used drug chemical substructures and drug targets as features, and “Max" as the similarity integration method to measure drug similarities. We compared the performance of our method with the state-of-the-art methods reported in [20]. As our method is binary-classification based, we just report metrics which are designed for binary classification. The results are listed in Table 3 and the best performance of a given metric is highlighted in bold values. Table 3 shows that our method outperformed all other methods in terms of AUC-PR (area under the precision-recall curve). In addition, our method achieved comparable average precision and AUC-ROC (area under the receiver operating characteristic curve) with the best performance (0.5439 vs 0.5476 in average precision, 0.9086 vs 0.9091 in AUC-ROC). The results further confirm the predictive power of the proposed method.

**Table 3:** Performance of the proposed method and state-of-the-art-methods using 5-fold cross-validation on Liu’s data set

Method	Average precision	AUC-ROC	AUC-PR
Our method	0.5439	0.9086	**0.5424**
Liu’s method [31]	0.2610	0.8850	0.2514
FS-MLKNN [32]	0.5134	0.9034	0.4802
LNSM-SMI [32]	**0.5476**	0.8986	0.5053
LNSM-CMI [32]	0.5329	**0.9091**	0.4909
KG-SIM-PROP [44]	0.4895	0.8860	0.4295

The best performance for each evaluation metric is showed in boldface

### Drugs that have the predicted side-effect “drug eruption (c0221242)”: a case study

This section presents a list of drugs which are predicted to have the side-effect “drug eruption” by the proposed method. Drug eruption is a side-effect on skin [33]. Most drug-induced eruptions are mild and they can disappear after stop taking the drugs. However, serious drug eruptions sometimes are associated with organ injuries like kidney and liver damage. It has been estimated that every 2-3 in 100 hospitalized patients have been suffering a drug eruption, and serious drug eruptions occur in around 1 in 1000 patients [34]. Consequently, to predict potential drugs which could possibly lead to drug eruptions is of great interests.

**Fig. 7:**
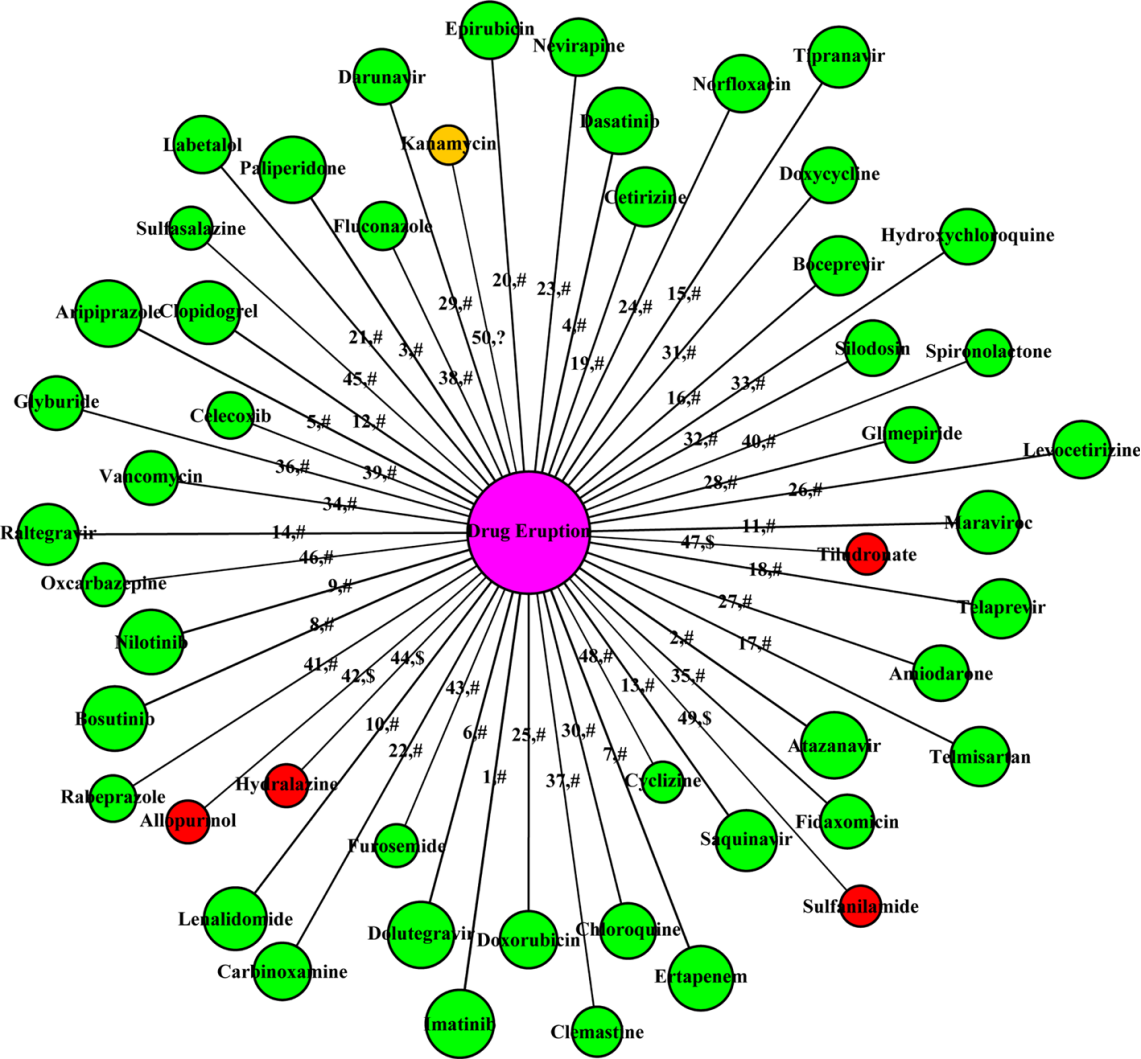
The top 50 drugs which are predicted to have the side-effect “drug eruption”. Labels on the edges illustrate the rank of predicted associations and the confirmation types

We performed the prediction using KNN (*k*=50) with validated positive samples and reliable negative samples selected by the proposed negative sample selection method. Like other data mining results, it is unrealistic to expect every predicted drug is of value to domain experts [35]. Therefore, we shortlist the 50 top-ranked drugs in terms of their prediction scores in Fig. 7. The circle in the center of the figure is the side-effect “drug eruption” and the other circles are the top 50 drugs. Among the top 50 drugs, the larger the prediction score is, the larger its circle is. Besides, the labels on the edges show the ranking positions and evidence types of the predicted associations. The symbols “#” and “$” denote that the corresponding associations can be validated by records from the side-effect database SIDER (colored green) and related literature (colored red) respectively. The symbol “?” means the predicted associations cannot be validated to the best of our knowledge (colored orange). Overall, 49 of the top 50 predicted associations can be verified by the SIDER and other literature (SIDER: 45, other literature: 4).

Drugs including allopurinol (DB00437), hydralazine (DB01275), sulfanilamide (DB00259), tiludronate (DB01133) and kanamycin (DB01172) are newly predicted (i.e., not stored in SIDER) to be associated with “drug eruption”. 4 of the above 5 novel associations can be confirmed by the literature work. Allopurinol is a medication used to decrease high blood uric acid levels [36]. In 2012, Kim et al. [37] studied its relationship with fixed drug eruptions using a lymphocyte transformation test. Fixed drug eruption is a distinct type of drug eruption which occurs in the same skin area each time when the patients take the drug [33, 38]. They finally confirmed that allopurinol is one of the causative drugs that induced fixed drug eruption [37]. Hydralazine, a well-known antihypertensive drug, has been in vogue for the last three decades. It was reported to induce fixed drug eruption in literature [39]. Sulfanilamide, a sulfonamide antibacterial, was widely used by the Allies in World War II to reduce infection rates. It contributed a lot to reducing the mortality rates. Early in 1939, Loveman et al. [40] reported fixed drug eruptions and stomatitis due to sulfanilamide. Tiludronate is a bisphosphonate used for treatment of Paget’s disease of bone. Its association with drug eruption can be found in the 22nd edition of Litt’s Drug Eruption and Reaction Manual [41].

To further demonstrate the capacity of the proposed method in predicting new DSPs, we also investigated those drugs which are ranked from top-51 to top-60. Among them, triclosan, nitric oxide, carbimazole, propylthiouracil, neomycin and dapsone are newly predicted drugs to cause “drug eruptions” and require further validation. Such newly predicted associations may provide interesting information for domain experts. In summary, the above successful prediction instances further demonstrate that our method has the capacity to predict both existing and novel drug-side-effect associations.

## Conclusions

In this work, we proposed an improved drug side-effect prediction method by selecting highly-reliable negative samples using a inverse similarity hypothesis and a new drug similarity integration framework. This framework captures drug similarity information from several aspects, including drug chemical structures, target proteins, drug substituents, and drug therapeutic data. Unlabeled drugs were preferably selected as negative samples according to their dissimilarities to labeled drugs. We adopted both the hypothesis of existing prediction methods, that similar drugs are more likely to share the same side-effects, and its inverse proposition. Thus, predictions using our highly-reliable negative samples rely on the validated positive samples as well as the selected negative samples. The originality of the proposed method lies in the negative samples selection, and in the prediction of a huge quantity of potential drug side-effect associations at a time. In the cross validation experiments, all results show that the prediction performance improved significantly using our method. The case study about drug eruption indicates that our method is capable to predict both existing and novel drug-side-effect associations.

Our method is useful in various areas and able to guide the drug development at different stages. For instance, at the early stage of drug candidate selection, our method can help to decide whether the drug molecules should be dropped or kept for further study. Our method can also help to find new indications of drugs, a process called drug reposition which could reduce both the time and financial cost of drug development largely [42, 43]. In addition, warnings about the potential side-effects of certain marketed drugs can be given to the public on time.

In this work, chemical structures, target proteins, drug substituents, and drug therapeutic information were integrated in a unified framework to predict side-effects. It should be pointed out that more drug data, e.g., drug chemical formulas, can be integrated into this framework. One limitation of the proposed method is its high dependence on the availability of drug chemical structures, target proteins, drug substituents, and drug therapeutic data, which are not always available and complete for many drugs. Consequently, the development of methods to enrich drug features from heterogeneous data sources is our future work. As addressed in Muñoz’s work [20], knowledge graphs which provide easy and automated integration of multiple diverse data sets in a uniform representation will be an ideal choice. In addition, modeling the side-effect prediction as a multi-label learning problem and making full use of off-the-shelf algorithms provide opportunities for us to further improve side-effect predicting.

## Additional files

Additional file 1 Supplementary codes and data. The two datasets used in this work and the python codes of the proposed method. (ZIP 11,623 kb)

Additional file 2 The supplementary results for this work. This file contains lists of researched drugs and side-effects, and all prediction results. **Table S1**: List of 917 drugs studied in this work. Drug names, drug target proteins, substituents of drugs, ATC codes and the SMILES strings are included as well. **Table S2**: List of 500 side-effect terms studied in this work. **Table S3**: *F*1-scores of different similarity measurements and different similarity integration methods using KNN. **Table S4**: *F*1-scores of different classifiers using the comprehensive similarity on balanced and imbalanced training sets. **Table S5**: *F*1-scores of different classifiers using the proposed negative sample selection method and random sample selection method. (XLSX 857 kb)

Additional file 3 The supplementary figures for this work. **Figure S1**: Scatter plots of *F*1-scores for different classifiers using the ChemTar similarity on balanced and imbalanced dataset. **Figure S2**: Scatter plots of *F*1-scores for ComNegative and ChemTarRandom using different classifiers. (PDF 5416 kb)

## Abbreviations

ATC: Anatomical therapeutic chemical
CCA: Canonical correlation analysis, CDK: Chemistry development kit
ELM: Extreme learning machine
GO: Gene ontology
KNN: K-nearest neighbors
RBF: Radial basis function
SCCA: Sparse canonical correlation analysis, SVM: Support vector machine
