# Supplementary material for: Inverse similarity and reliable negative samples for drug side-effect prediction
Source: BMC Bioinformatics. 2019 Feb 4;19(Suppl 13):554. doi: 10.1186/s12859-018-2563-x (PMC7402513; doi:10.1186/s12859-018-2563-x)
Supplement: Supplementary file 3 — The supplementary figures for this work. Figure S1: Scatter plots of F1-scores for different classifiers using the ChemTar similarity on balanced and imbalanced dataset. Figure S2: Scatter plots of F1-scores for ComNegative and ChemTarRandom using different classifiers. (PDF 5416 kb) [file 12859_2018_2563_MOESM3_ESM.pdf]

Figure S1

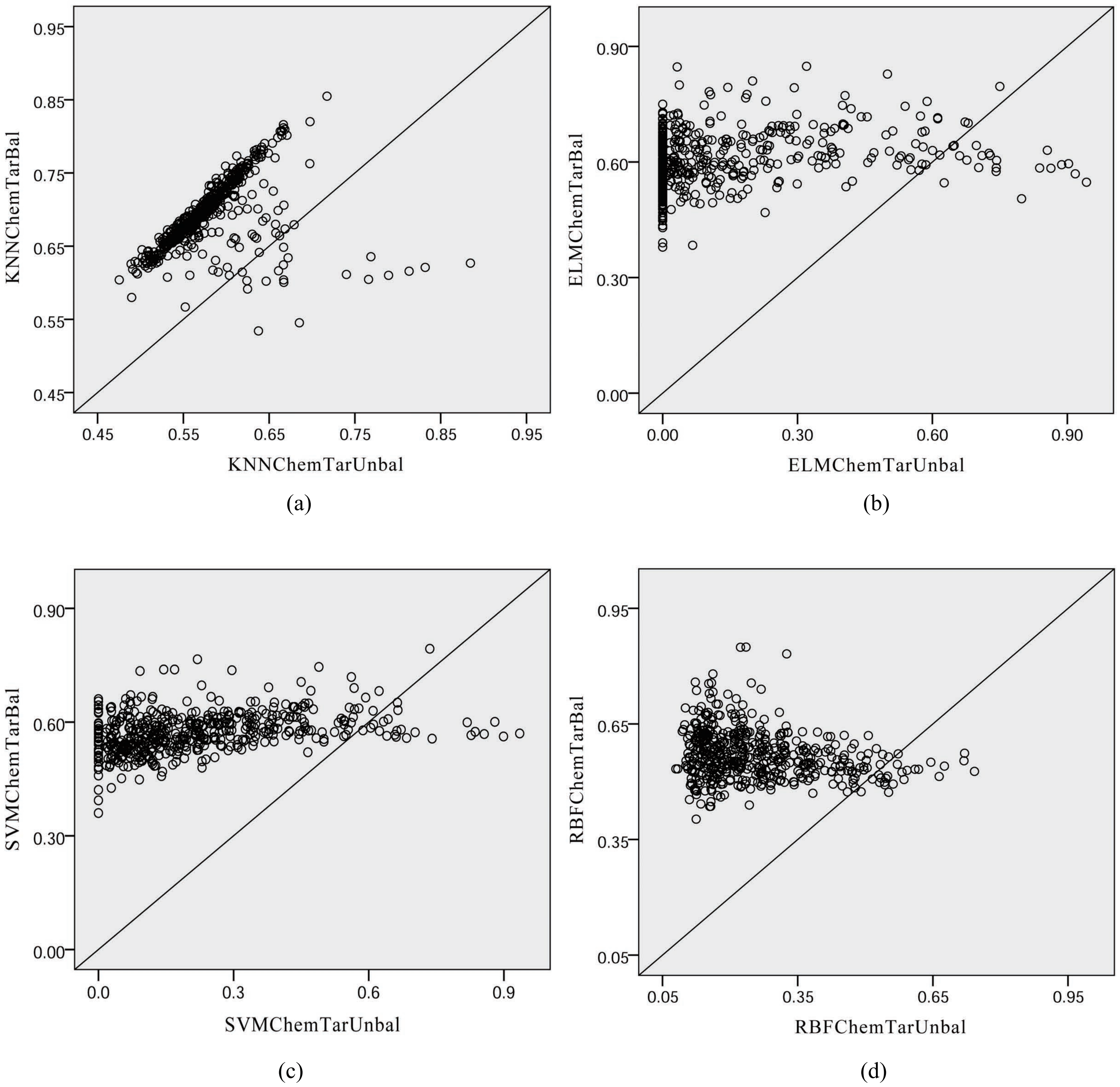

Scatter plots of F1-Scores for different classifiers using chemTar similarity on balanced and imbalanced datasets.

Figure S2

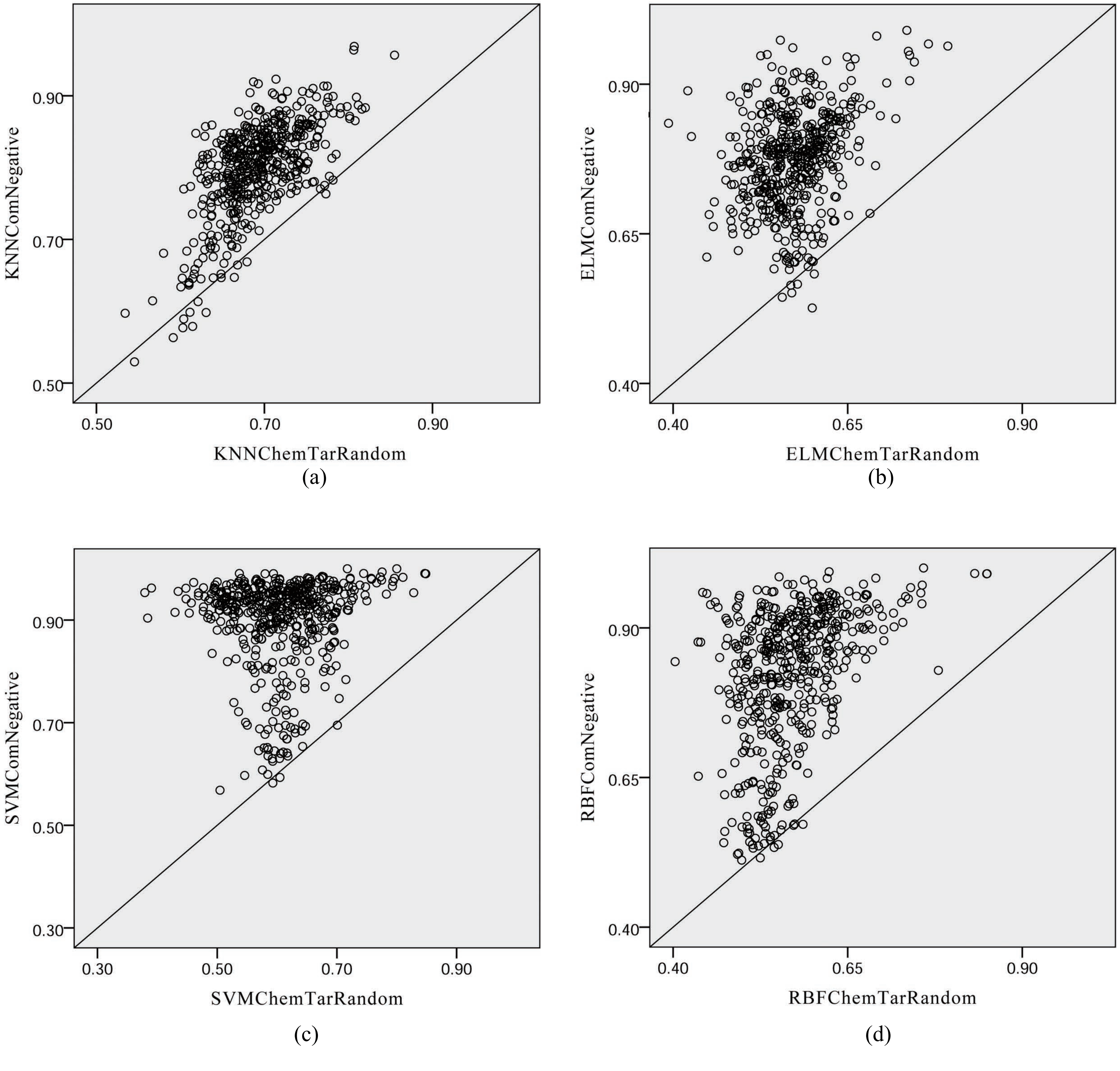

Scatter plots of F1-Scores for comNegative and chemTarRandom using different classifiers.
